# Familiarity and participation outside home for persons living with dementia

**DOI:** 10.1177/14713012211002030

**Published:** 2021-03-27

**Authors:** Isabel Margot-Cattin, Nicolas Kühne, Annika Öhman, Anna Brorsson, Louise Nygard

**Affiliations:** Department of Occupational Therapy, School of Social Work and Health - Lausanne (HETSL), 111832 University of Applied Sciences and Arts of Western Switzerland (HES-SO), Switzerland; Division of Occupational Therapy, Department of Neurobiology, Care Science and Society (NVS), Karolinska Institute, Stockholm, Sweden; Department of Occupational Therapy, School of Social Work and Health - Lausanne (HETSL), University of Applied Sciences and Arts of Western Switzerland (HES-SO), Switzerland; Unit of Occupational Therapy, Department of Health, Medicine and Caring Sciences, 4566 Linköping University, Norrköping, Sweden; Division of Occupational Therapy, Department of Neurobiology, Care Science and Society (NVS), 27106 Karolinska Institute, Stockholm, Sweden; Division of Occupational Therapy, Department of Neurobiology, Care Science and Society (NVS), 27106 Karolinska Institute, Stockholm, Sweden

**Keywords:** familiarity, dementia, territories, places, participation outside home

## Abstract

Familiarity is important for persons living with dementia who participate outside home. When familiarity is challenged, such participation may be difficult. This ethnographic study clarifies how familiarity is experienced by persons with dementia in performing activities and visiting places, and how familiarity contributes to maintaining participation outside home. Nine participants were interviewed in their home and while visiting familiar places. Data were content analysed using a constant comparative method. The findings suggest that persons with dementia experience familiarity as continuous and whole, through occurrences that support personal territories. Landmarks and objects enhance the experience of familiarity. Familiarity that is continuously challenged may render participation outside home fragile.

## Introduction

In Switzerland, 151,000 persons are living with dementia, of which about 80% are living at home, with support of home health and community services (Association Alzheimer Suisse, 2018; Office fédéral de la santé publique (OFSP), 2019). Dementia affects an individual’s capacity to engage in everyday activities and participate in society at home and outside the home, where participation is often described as a shrinking world (Duggan et al., 2008). Historically, participation has been developed and used in the disability movement as a political term to describe human rights for non-discrimination (United Nations Convention on the Rights of Persons with Disabilities (CRPD), 2007). More recently, participation has been defined from an individual perspective, mostly seen through the International Classification of Functioning, Disability and Health (ICF) (World Health Organisation, 2001) definition: ‘involvement in a real life situation’ or a level of performance in activities (Velde et al., 2018). However, participation is more than the quantifiable performance of an activity (Martin Ginis et al., 2017), and includes agency, engagement, purpose, meaning and satisfaction and acquiring skills. Conceptualisation of participation as essential for health and well-being requires a perspective extending beyond how a person performs in an activity, and takes into account experiences lived while so engaged. The wish of older adults to ‘age in place’ has highlighted participation outside home for persons with dementia (Rowles & Bernard, 2013) as an understanding of experiences when facing changes and challenges in accessing places and activities.

### Defining familiarity

Familiarity is important in supporting people to participate outside home (Bontje et al., 2019; Brorsson et al., 2016; Malinowsky et al., 2019). In neuropsychology, familiarity is a memory process, different from recollection, based on assessments of stimuli, like recognising a person, a place or event as having been seen or experienced before (Yonelinas, 2002). Stepping away from considering familiarity only as a cognitive function, Philips et al. (2013) consider how familiar environments might become unfamiliar due to urban renewal or cognitive decline. Experiencing non-familiarity may lead to insecurity and disorientation, fear about personal safety and social exclusion and a decrease in participation outside home.

Fear of getting lost and being embarrassed deters persons with dementia from going to previously familiar places that now feel unfamiliar (Phillips et al., 2011). Therefore, maintaining familiarity by regularly and repeatedly visiting the same places enables navigation in surroundings close to home (Brorsson et al., 2011; Phillips et al., 2011), by helping individual landmarks to emerge from the repeated experience of ‘getting there and back’ (Seetharaman et al., 2018). Repeated and frequent visits help keep landmarks and places familiar through procedural memory (Bier et al., 2015; Zanetti et al., 2001), as familiarity is triggered without conscious thought to navigate to places (Mossabir, 2018).

However, familiarity is not just attached to the environment or places but includes occupations performed in places regularly visited, creating what is called ‘familiar lived and practiced places’ (Mossabir, 2018). Furthermore, it is possible to consider persons as constituents of places where they live and practice – in transaction – highlighting a person–environment relationship that supports the idea of embedded beings in familiar places (Andrews et al., 2013; Cresswell, 2015). From a relational, transactional perspective, people engage in activities in accordance to their values, to support their need for continuity in life, and thus create meaning and give them goals (Cutchin & Dickie, 2013). People experience being in a familiar place, which includes having an affective (attachment) and cognitive (identity) link to that place (Meijering et al., 2019). Activities performed in familiar places are situated and embedded, supporting the link between a person and a place (Margot-Cattin, 2018). As such, familiarity can be better understood as a situated, embedded and practiced experience of embodied places to which persons with dementia travel (Kontos & Martin, 2013; Seetharaman, 2018).

### Visiting places outside home

Recent studies Margot-Cattin et al., 2021; Gaber et al., 2019) add to the idea that people with dementia face changes and decreases in participation outside home (Duggan et al., 2008) in a way that is not even, clear and linear. In fact, there seems to be an increase in the use of medically oriented places like day hospitals or clinics, to the detriment of cultural and social places like concert halls or association venues. There is also an indication that older adults with dementia abandon more places and to a larger extent than their peers without dementia, and that familiar places – the neighbourhood or family member’s house – might remain familiar longer (Gaber et al., 2019).

In sum, familiarity is an important but understudied aspect in support of persons with dementia maintaining participation outside home by navigating and reaching places, and engaging in activities. The familiar becoming unfamiliar may restrain people with dementia from such participation. Familiarity is also challenged by recurrent urban renewal and a progression of cognitive decline in early-to-medium stages of dementia. However, there is a knowledge gap in how people with dementia experience familiarity in places and activities, how they cope with losing familiarity and how familiarity or unfamiliarity may contribute to participation outside home.

The study aim is to clarify how familiarity is experienced by persons with dementia performing activities and visiting places outside home, including describing how they experience the characteristics of familiarity, and how familiarity might contribute to maintaining participation.

## Methods

### Conceptual frame

This ethnographic study reports on research undertaken as part of the ‘life outside home for people with dementia’, an international project lead by Karolinska Institutet in Sweden^1^. This article presents and discusses findings from the qualitative part in the French-speaking field site in Switzerland.

### Participant recruitment and setting

Recruitment proceeded via memory clinics, day-care centres and the Alzheimer Association. Inclusion criteria were as follows: over 65 years old, a community-dwelling adult with a dementia diagnosis from a memory clinic and the capacity to give informed consent (Dewing, 2007). We used the person-centred process consent method, enabling researchers to include consent communicated through behaviour and non-verbal means by persons with dementia (Dewing, 2002, 2007). Ongoing consent monitoring (observing signs of stress, anxiety or discomfort) was applied throughout the data collection process to ensure that no undue stress or burden arose from participating in the study (McKeown et al., 2010). Continued assent was also assessed by observing signs of discomfort, confusion or unease. If signs of discomfort were observed, we took a break to discuss them and decide together how to proceed.

Significant others were present when needed to provide support during the home-based interview, as people with early-to-moderate stage of dementia generally are able to respond to clear and unequivocal questions, in a familiar setting (Nygård, 2006). Participants were informed about the aims of the study orally and in writing, and gave signed consent to participate. In Switzerland, persons living with dementia may give informed consent as long as they are not under a guardianship, which is dependent on a legal decision. The significant others of participants were also informed orally and in writing. Ethical authorisation (protocol 452/15) was obtained from the “Commission cantonale d’éthique de la recherche sur l’être humain (CER-VD)” in Lausanne, Switzerland.

We recruited nine participants (Table 1.) in a convenience sample. The participants were at various stages of dementia, as denoted by the MoCA score (9–27). For anonymity, participants’ names were changed. At the time of the study, four men out of five lived with their spouses, while all women lived alone. All participants spoke French as a first or second language, as they had been living in the French-speaking region of Switzerland for most of their life.

**Table 1.** Participants’ characteristics.

Participant (not real names)	Age	Sex	Type of dwelling	Year in the dwelling	Urban/rural	MoCA score^1^	Sig. other present 1st interview
Edith	65	F	2 apartment house	40	Urban	17	No
Georges	74	M	Apartment block	50	Urban	16	Yes
Charles	71	M	Individual house	30	Rural	21	Yes
Henri	65	M	Individual house	35	Rural	21	No
Samuel	69	M	Individual house	15	Rural	24	No
Fanny	85	F	Individual house	50	Urban	27	No
Anita	72	F	Apartment block	48	Urban	20	No
Paul	82	M	2 apartment house	62	Rural	19	No
Rose	90	F	Apartment block	25	Rural	9	No

1 The MoCA (Nasreddine et al., 2005) score is given here as an indication of cognitive level, higher score indicates higher cognitive function (maximum 30).

### Data collection

Data collection constituted two types of interviews: a two-phase sit-down, face-to-face, home-based interview (Keady et al., 2018) and a walk-along mobile interview done outside the home (Carpiano, 2009), while the person was going to familiar places and performing activities. We included mobile interviews to allow participants to comment on their enacted situated experiences of familiarity (Mossabir, 2018). Mobile interviews provided knowledge on embodiment and meaning of familiar places and familiar activities, well aligned with the theoretical perspective of this study (Clark & Emmel, 2010; Kullberg & Odzakovic, 2018; Merriman, 2014). This method allowed researchers to be part of the experience while walking along with the participant. It supported the creation of rich data in an innovative interview process (Carpiano, 2009; Kinney, 2017; Odzakovic et al., 2018), as walking, talking and being in places outside home generated data about familiarity within the enacted experience of the transactional situation (Hand et al., 2017).

Data collection was conducted by the first author and audio recorded. The home-based interview aimed to exchange information about the study (aims and roles) and our mutual involvement, to build trust, and to get an impression of the person’s situation. Questions in the interview guide were about participants’ interests, routines, activities, and participation outside home and about familiar places. The first (home-based) interview lasted between 60 and 90 min. We invited significant others to stay for the first interview; Georges’ wife stayed for the whole interview and Charles’ wife was present only for the first 30 min. Other participants did not choose to have a significant other take part. At the end of the home-based interview, we discussed where we would go for the mobile interview in the next few days or week. We conducted from one to three mobile interviews with the participants, adding up to a total of nine home-based interviews and 15 walking interviews.

For the mobile interviews, participants chose where they wanted to go, with the condition that it be a familiar place, part of their habits or routines. The aim of walk-along interview was to get participants to talk freely about ongoing experiences of familiarity, while taking the first author to familiar places. The interviews were open-format informal conversations, creating a relaxed, friendly atmosphere. Direct questions were asked about places (e.g. why did you choose to take me here? What makes this place familiar to you?), finding one’s way (e.g. how do you know where you are?) and activities performed (e.g. what do you usually do here? How do you understand this place as familiar?). Participants often pointed to specific landmarks, or told stories about places, as if they were a ‘tour guide’. We stopped to discuss experiences and feelings of being in or going to familiar places, with probing questions regarding the experiences (Burgess, 2000; Hammersley & Atkinson, 2007), such as ‘Please, tell me more about that experience’, or ‘how would you describe that feeling?’ Some mobile interviews were short (30 min), while others lasted up to 150 min. We went to many different places: the pharmacy (for a prescription), churches in the mountains to take pictures (for a participant to write an article for the village journal) or a hike in the forest to collect mushrooms. Mobile interviews took place in all seasons, during the day, and in any weather, rain and snow included. After each home-based interview and mobile conversation, the first author wrote memos about the settings, state of mind, and reflections about the interview itself, which were included in the data to inform the analysis (Hammersley & Atkinson, 2007). Both types of interview recordings were transcribed verbatim by a certified transcriptionist; these transcripts were checked by the interviewer (the first author).

### Data analysis

The analysis was performed parallel to data collection in this ethnographic research process (Hammersley & Atkinson, 2007; Nayar & Stanley, 2015). The transcribed interviews and field notes were gradually added to NVIVO software (v 11.4.3).

Transcripts and field notes were read several times to immerse oneself in the participants’ experiences and get a grasp of important aspects and feelings generated (Boeije, 2002; Bryman & Burgess, 1994). Initially, the first, second and last authors used a constant comparison method to highlight words and sentences as focal points of the experience of familiarity (Charmaz, 2014). Codes were used to describe contents, for example, the feelings associated with familiarity or how familiarity was characterised. Codes were compared, matched and opposed. Tentative themes began to emerge like how the idea of familiarity might be challenged, with codes of ‘risks’, ‘disturbing events’, ‘strategies for familiarity’, ‘familiarity and risk relations’ and ‘going out’.

We used an iterative process to compare emerging themes with the theoretical framework of this study (familiarity, person–environment relationship and out-of-home participation), thus enriching the analysis (Hammersley & Atkinson, 2007). To refine our interpretations, ideas of themes were contemplated using a transactional perspective and theories of place (Cresswell, 2015). The idea of territories being created from ‘pieces’ of fluctuating familiarity (places and activities) emerged from the comparison. Paquot’s (2011) input from ethology and social anthropology (when a predator appears, the territory becomes smaller) highlighted that persons with dementia are in constant relation to their personal territories and take part in construction of those territories (Carpenter, 1958; Malmberg, 1980). The idea of landmarks and objects serving as lifeline was better understood through the theory of saliency (landmarks) (Seetharaman, 2018) and ‘being in place’ (Rowles & Bernard, 2013). Theory on embodiment of objects (Cresswell, 2015; Quinton, 2019) and the idea of continuity from the transactional perspective (Margot-Cattin, 2018; Rowles & Chaudhury, 2005) were used to support understanding the roles of individual objects in experiences of participation outside home. As participants also described or enacted situated experiences in which familiarity was at risk, we identified those and reflected on their contribution to the potential loss of familiar places (Phillips et al., 2011). To illustrate our analysis, we translated and cited fragments of data from French to English.

## Findings

The findings are presented in three themes: (a) *familiarity is experienced in a continuous way, as a whole and through occurrences*; (b) *familiarity is enacted through a personal territory* and (c) *landmarks and objects in the experience of familiarity*. The first theme explains how participants experience familiarity; the second explains how the experience of familiarity is maintained in participation outside home, by enacting it in a territory and the third explains the role of landmarks and objects. Although the themes stand alone, they are experienced by participants simultaneously in the situations they live.

### Familiarity is experienced in a continuous way, as a whole and through occurrences

For participants with dementia, familiarity was an embedded part of their everyday activities outside home. They took familiarity for granted and continued out-of-home activities without consciously thinking about it. They walked streets or paths, took turns and did their usual activities in places they reached. Their experience of familiarity was implicit, obvious in a way that had participants say: ‘Of course it’s familiar’. Familiarity was embedded in everything they did and everywhere they went, offering a continuous experience of participation outside home. A ‘continuous experience’ here relates to the concept of continuity (Dewey & Bentley, 1949): identifying oneself as a continuous sensation of experience in relationship to ‘an organism in an environment as a whole’. Time passes, conferring a past, present and future to the person. So, the experience of familiarity is continuous and is qualified as a ‘whole’ by participants. Furthermore, they described familiarity in diverse ways, bringing personal meaning to qualify it, linked to their specific understanding and situation. For Fanny, familiarity was knowledge: ‘*I know my city*’; for Edith, it was part of a routine: ‘*It is something so usual that it is an automatism*’; for Henri, familiarity provided a sense of security, social belonging and well-being: ‘*It’s super familiar, I’m safe, people are nice to me and I’m good*’ and Paul expressed it as a cohesive whole: ‘*It’s a whole, not pieces*’. The metaphor of a ‘whole’ to qualify familiarity does not mean it was homogenous. A ‘whole’ could include various items, ingredients or elements, like a hamburger that would be eaten whole; it contains meat, bread, cucumber and sauce, but is one complete object.

Interestingly, this idea of familiarity being metaphorically qualified as a ‘whole’ is reported by other participants through stories. They tell about the places we visited, grounded in their personal and familial histories; they include other persons, family members or friends. These stories include moving to and reaching the peculiar locations – ‘getting there’ – and doing specified activities, making it a story about a ‘whole’. The stories were embedded in the places, accentuating the relation between the person and the place, in situations and ‘whole’. For them, stories gave meaning to places and supported their experience of familiarity. When Edith went shopping in the city, we stopped at a café and she told that she would always expect to meet her sister-in-law in that place. That café had been a rallying place for the two sisters-in-law for decades and was part of their twice-weekly routine: ‘*But hey, I go to the cafe, and then I go shopping, and then I go home to cook*’. Edith was very clear about her shopping routine. Storytelling was triggered by being and doing in that same place; it included the narrator embedded in the place (café in the city), time and continuous experience (identity), so that she would always recognise that place (in her discourse). The familiar place (café) was thus narrated, historicised and included other persons (sister-in-law). The experience of familiarity is situated in the place and as such is part of experiencing the place as a ‘whole’, supporting the feeling of continuity of one’s own identity, as described in the transactional perspective theory.

Furthermore, participants experienced familiarity as situations were repeated over time, that is, going to the same place repeatedly, like grocery shopping in the same store. Each occurrence of the same repeated situation would be slightly different, for example, due to the weather, the ingredients needed to be bought or being accompanied by a family member. Everyday life situations would thus be specific and unique for the participants, and repeating the same situation supported the sensation of continuity. So, participants found themselves in repeated one-time occurrences holding various features that over time built a recognition of the place as familiar and whole, supporting a continuity of experience. Again Edith said, as we strolled the streets: ‘*No, I cannot get lost, it’s in my genes! I was born in this city*’. Although there were roadworks in a street close to her home, she did not falter, continuing as if this was not an issue, just a feature that can change in a high number of occurrences she has used to build the experience of familiarity in her city. Other features of those occurrences would change – like the weather (snow or ice in the street), time of the day and a different grocery cashier – sometimes bringing challenges to the experience of familiarity. Anita reported a challenging experience in one occurrence: *‘When it’s raining, it’s all gray and blurry so I’m not sure where I’m going. Once, I went out, it was not for long, it was cold and wet, I could not see where I was going, so it was a friend with her dog who found me and accompanied me back home*’. When the streets were under construction, Charles, who was going to the barber shop up the street, ended up at the grocery store because he had to take a detour to circumvent the roadworks, and forgot where he was initially going. Again, Anita, who regularly went to her familiar grocery shop, experienced an embarrassing experience when she put more items in her cart than she had money to pay for. In this occurrence, when she arrived at the counter, the usual lady was not there: ‘*I cried at the counter, because I didn’t have enough money. […] And it was an unfamiliar person at the counter, but she helped me get everything out of the cart. It was so embarrassing*’. In these examples, the experience of familiarity was dependent on specific situations, and familiarity may fluctuate over time and occurrences.

### Familiarity is enacted through a personal territory

As presented above, participants experienced repeated one-time occurrences with various features in building familiar places that were recognised as such. In their experiences, these familiar places are not separated from one another, but rather form territories in which participants may safely navigate, participate and do activities, without getting lost or embarrassed. These territories, specific to the individual, could be seen as personal. The successions of places and activities in the personal territory are repeatable one-time occurrences, enacted by participants in everyday life, and holding individual meaning. For example, David was used to doing a lot of sports and he needed to exercise everyday: ‘*Yes, I will run in the forest or ride a bike. In fact, I’m always outside for hours. My territory is not just around the house, it’s the forest and beyond, to the river [name of the river]. Since they took away my driving license, I bike instead*’. David explained that he had always been very active outside, that it was part of his identity and his way of participating, while we walked in his forest instead of running. David referred to those familiar places as ‘his’ territory that he was able to define, naming the river. Experiencing his territory as familiar supported his participation outside home, allowing him to walk, run or bike through it, without fear of getting lost, as long as he stayed in his personal territory.

Sometimes, participants may even take the risk of getting lost in that territory. Edith said she has travelled in the city (her familiar territory) every day for more than 30 years: ‘*I can find myself all of a sudden in a place that will be …, all of a sudden I don’t know where I am and well I say “I’m lost” but then I think a little, I look a little on the right, I look a little on the left, and so on, but yes, suddenly I see where I am, I have seen a landmark and that’s it*’. The personal territory was understood here as being an agglomerate of familiar places that not only belong to them (making it personal – ‘my territory’) but also to which they belong, in a transactional view of people and places, co-constructing each other in the experience of familiarity. Thus, familiarity was continuously experienced by participants as a personal ‘whole’ territory made of familiar places, enabling participants to move inside their territory in a fluent, flowing and safe manner, while still able and supported in taking risks.

As seen before, the experience of familiarity was challenged in some occurrences due to features in the situation that unbalanced the participant’s experience. These challenges created temporary fluctuations in an otherwise familiar territory. For example, Henri had to stop hiking in the mountains during the winter, a lapse of use. The place had become unfamiliar in winter, and he had to ask his wife to go with him the first time he went back to find the chalet where he starts hiking: ‘*I need to make an effort, … sometimes my wife helps me and comes with me the few first times I go back hiking. I don’t remember which chalet I need to drive to, they all look the same in the spring, but then when I get there, after a couple of times, I get this feeling of rightness, of familiarity, and I can start hiking again*’. Changing features in familiar places, like roadworks, time of no-use or any difference in occurrences could unbalance a participant’s experience of familiarity. These disturbances in the experience of familiarity might be linked to the vulnerability of persons with dementia in maintaining participation outside home. It might also support a participant’s sensitivity to changes in familiar places.

Furthermore, as expected, the familiar personal territory for persons living with dementia might be the neighbourhood, in walking distance to the house. But for some participants, we went further away, either driving or walking. Henri, who lives in the Alps, wanted to write an article in his village journal on churches in the surrounding mountains. So, he drove to seven churches to take pictures; when there, he told us the stories he wanted to write about. Henri had regularly visited these churches as a boy, hiking with his family and as a retiree. These churches were familiar, but well outside his direct neighbourhood. The same can be said for Samuel, whose family has owned a patch of forested land for a long time. He used to run, bike and hike in that forest, as we did together: ‘*Oh yes, I am free in the forest, and if I lose myself, it does not matter, I come out on one side and I find my way. My family has always had this forest, I grew up there, so ..*’.^2^ These examples argued for understanding the experience of familiarity through the representation of personal territory, where there might be familiar places within and outside of the neighbourhood. As such, familiarity does not equate proximity.

### Landmarks and objects in the experience of familiarity

Some environmental features were recognised by participants as reference points, like landmarks or spatial anchor points. Some participants used familiar landmarks, like Henri with the chalet where he parked to go hiking: ‘*Here I go to the chalet we see there, by car, I park in front of ... .ah I don’t always know the names of the chalets, but I park and then I walk on the paths in the mountains, that’s my life ... sometimes my wife comes with me, but often she lets me go alone. The mountain is beautiful, from the spring, I go to walk*’. The chalet acted as an anchor, a familiar reference he knew to return to.

Charles regularly went walking in and around his village, going from one fountain to another, where the clearest and freshest water was. He always took a flask with some absinthe^3^ for his walk, to meet friends while touring the fountains. He took his familiar flask on all his outings, even if not touring the fountains, explaining that carrying a familiar object acted as a lifeline to his home. The flask enabled Charles to experience reassurance and safety while navigating his personal territory. Other participants had familiar objects with them: Edith took her keys, although she never locked her door; Samuel took his wallet and a bottle of water, also when he went with his wife to the grocery store and Anita carried an umbrella when it was not raining. Although some objects made sense with the activity that participants undertook – like taking a bottle of water to go hiking – the same objects were taken even when they were not needed. These objects could then be understood as being taken for more than practical reason; by being familiar, they helped in participation outside home.

## Discussion

This study sought to clarify how familiarity might be experienced by persons with dementia and give light to how familiarity might contribute to maintaining participation while visiting places where occupations are performed outside home. The findings suggest that people with dementia experience familiarity as continuous, through occurrences that sustain familiarity in personal territories with facilitating landmarks and objects. In fact, places are constantly shaped by how people interact and actively engage in activities (Clark et al., 2020). As Andrews et al. (2007) stated, place is too frequently considered as a container, instead of the result of interactions between the person and the environment. Considering places as being co-constructed has been supported by social anthropology (Carpenter, 1958; Malmberg, 1980) for some time, but the role of familiarity has not been explored. Our results point to a relationship between familiarity and the construction of personal territories, as experiencing familiarity might be necessary for our participants’ creating and using territories (Fung, 2020; Hay, 1998; McGovern, 2017). Territories seem to be constructed at the crossroads between environment (physical territory), social relations (social territory) and given meanings (cultural territory) (Sack, 1992). Human relationships to territories hold emotional, cognitive and functional aspects. They are not fixed in time but evolve and need to be continuously individually ‘negotiated’, meaning that places need to be visited and ‘used’ by performing activities (Bontje et al., 2019). Performing activities in places creates a structure, repeated occurrences and rhythms, predictability and patterns that contribute to familiarity (la Cour et al., 2009). Thus, a continuously ‘negotiated’ territory is experienced as familiar, as our participants have described; familiar places are considered as a meaningful ‘whole’, including transportation (walking, public transport or driving), performing activities, relationships and co-construction (Meijering et al., 2019). Personal territories for persons with dementia constitute familiar places they need to maintain by visiting and performing activities, making the issue of community mobility important. Finding ways of supporting familiarity, like regularly and frequently repeating the visits to important places in their personal territory (either alone or accompanied), might enable older adults living with dementia to ‘age in place’ in their community.

Many studies have considered participation outside home to be restricted little by little, over time, to eventually only cover the neighbourhood, for example, a familiar space in walking distance from home (Blackman, 2006). However, our analysis highlights that people with dementia may go to familiar places further away that are still part of their personal territories and to which they need to travel using various means. Negotiating a personal familiar territory might be essential for maintaining identity. Echoing Clark et al. (2020), we suggest extending the concept of neighbourhood not just as a close by geographically limited space (within walking distance) but – in our interpretation – as territories in which people may actively engage in relation to their environments. In that way, support implemented in dementia-friendly actions would be extended to places further away and be more in line with the needs of persons with dementia.

In addition, our results propose that territories hold familiar landmarks that support people with dementia to participate outside home (Phillips et al., 2013; Seetharaman, 2018). As landmarks become familiar through the process of replacement by physically moving from home to destination and back (Moser, 2009), persons with dementia need to continuously ‘negotiate’ their personal territory. However, familiar landmarks are also vulnerable, as they can vanish or be changed by roadworks or painting or renovation (e.g. for a mountain chalet). Choosing landmarks is subjective, dependent on saliency (visual, cognitive and structural) and needs a personal connection and an emotional response to be effective (Caduff & Timpf, 2008; Seetharaman, 2018). Saliency of landmarks is based on how well it contrasts with the context it is set in. Structures, urban furniture or arts tend to be landmarks of singularity and familiarity; clear form, visual or structural contrast enable a structure to be a landmark. Its saliency thus includes visibility, location, noticeability, identifiability, recognisability and memorability, attributes that increase through familiarity (Seetharaman, 2018). Our results underscore the challenges of creating and using landmarks in a territory to maintain familiarity. Being attentive to landmarks’ saliency might support participation outside home.

In our findings, familiar objects are more important than expected; bringing them out of the house might not be trivial, as they could create a lifeline between the familiar place of home and familiar places outside home, or be an anchor to home. Having familiar objects when going out could be necessary for practical reasons or from habit (a purse, a key to close the door and a cane to walk), but objects can hold other meanings and contribute to participation (Hocking, 1994; Marres, 2012). Our findings indicate that objects might hold meanings beyond those described earlier; this should be further explored. These findings might be used to support persons with dementia in maintaining an experience of familiarity outside home.

As explored before, participation outside home for persons with dementia can be fragile when familiarity is challenged. Familiar places are continuously changing due to how people interact with them, as they engage in familiar occupations outside home. Although we did not investigate potential relationships with cognitive function in this study, challenges to familiarity happened to most participants in diverse situations. Losing familiarity might be a common experience for persons with dementia, can happen suddenly, triggered by small changes in the environment (Brorsson et al., 2013) and is linked with experiencing risky situations (Sandberg et al., 2015). Our participants tried to cope with the challenge to familiarity by looking for a landmark or by resuming a routine, as also reported by others (Brorsson et al., 2013; Sandberg et al., 2015). Participants who experienced fluctuations in familiarity and tried to cope with it were at risk of withdrawing from participation outside home. Familiarity that supports their participation might be their opportunity to continue to be active and do meaningful activities.

### Study limitations

All participants had been living in the same housing for a long time (15–62 years). Mobile interviews were conducted in only familiar environments, as in the inclusion criteria. This may have partially limited the exploration of challenges to familiarity and how people with dementia cope in unfamiliar environments. All interviews were conducted in French, and the language criteria may have limited the scope of the results. Still, participants had a varied cultural background, as some immigrated when young, reflecting the state of the older population in Switzerland.

Although participants were living in familiar surroundings, they struggled to explicitly speak about familiarity. This might be due to familiarity being taken for granted, implicit in nature and enacted (Clark et al., 2020). Participants rather spoke of how they related emotionally to the places we visited and what their experiences were. This might also be explained by the difficulty of people with dementia to use spatial visualisation (Tucker-Drob, 2019) to answer interview questions. They might also have faced difficulties in verbalising their thoughts (which was required for this study), thus missing some potentially important elements of familiarity. Using mobile interviews, in which participants could enact their familiar environments, might have helped circumvent this difficulty.

Another limitation to this study is that the type and stage of dementia was not identified beyond the screening done by using MoCA. Collecting more information on the cognitive functional level and type of dementia of participants might have given a better insight on the difficulties that participants might have been facing during the interviews, especially with such a big range of MoCA score (9–27). Still, the aim of this study was to clarify how persons living with dementia experienced familiarity through their perception rather than focussing on familiarity as a cognitive construct.

## Conclusion

Participants with dementia experienced familiarity as continuous, a ‘whole’, expressed in personal territories with facilitating landmarks and objects. Still, familiarity may continuously be challenged, making participation outside home fragile. Even taking into consideration the complexity of each situation outside home, it is difficult to understand or predict how familiar places and activities become unfamiliar, as the relationship between the person and the environment changes constantly. This fluctuation might be pictured as a gap, a hole or a rift in familiarity, but characterising the experience is uncertain; this was not the focus of this study. More studies are needed to describe and explain the disruption of continuity in experiencing familiarity outside home.

## Notes

1 https://ki.se/en/nvs/participation-in-places-and-activities-in-public-space-as-perceived-by-people-with-cognitive.

2 Note that forests in Switzerland are small and trimmed by foresters. Paths are drawn and underbrush is not impassable. Many families in small villages own a patch of forest for harvesting wood, mushrooms, etc.… There are no wild forests in Switzerland, and there is only one national park where there is minimum human intervention.

3 Famous local alcohol, also commonly called ‘fée verte’ or ‘bleue’ due to its bluish colour when mixed with the freshest water, was illegal to drink and produce for decades. It has been legalised with less potency but is still partially produced illegally as locals want to keep its tradition.
